# Evaluation and Comparison of Rate of Canine Retraction Between Titanium Dioxide Nanocoated Stainless Steel Archwire Segments and Uncoated Stainless Steel Archwire Segments: An In Vivo Split Mouth Study

**DOI:** 10.7759/cureus.95012

**Published:** 2025-10-20

**Authors:** Padmashri Narayanan, Shailaja M, Pachaiyappan G, Karthika E.S

**Affiliations:** 1 Orthodontics and Dentofacial Orthopaedics, Sri Siddhartha Dental College and Hospital, Tumkur, IND

**Keywords:** canine retraction, nanocoating, space closure, stainless steel archwire, titanium dioxide

## Abstract

Objectives

Our objectives were to compare titanium dioxide (TiO₂) nanocoated stainless steel archwire segments with uncoated stainless steel archwire segments during individual canine retraction.

Materials and methods

A split-mouth study was carried out in vivo on 15 patients who needed bilateral maxillary canine retraction. One side of each patient's stainless steel archwire was coated with TiO₂ nanoparticles, while the other side was left untreated. Using nickel-titanium closed coil springs and 150 g of force, canine retraction was performed. Digital vernier calipers were used to take measurements at four-week intervals for 16 weeks.

Results

The rate of canine retraction was statistically significantly higher in the TiO₂ nanocoated archwire segments than in the untreated segments (p < 0.05). At the first, third, and fourth months in particular, significant differences were found (T1: p=0.021*, T3: p=0.021*, and T4: p=0.023*). There were no changes at baseline (T0: p=1.000) or during the second month (T2: p=0.126), suggesting that the beneficial effect of nanocoating became more pronounced as treatment progressed. Overall, the difference in total space closure was modest but statistically significant, with steady advancement of space closure over time.

Conclusion

TiO₂ nanocoating may enhance the efficiency of orthodontic tooth movement by reducing friction. In the present study, the nanocoated segment showed faster tooth movement compared to the uncoated segment.

## Introduction

In motion or attempted motion tangentially over an interface between two solid objects, a resistant force known as friction is produced. Its purpose is to dampen the relative motion or tendency for motion between two surfaces that are in touch with one another. A tooth can only be physically moved by applying force via its point of resistance equilibrium. But the tooth experiences both forces and moments since the force is usually exerted at the crown's bracket. One method employed for retraction of teeth is sliding mechanics. This technique involves sliding the archwire along the tooth's bonding bracket, which allows the tooth to move. The contact surface between the archwire and the bracket slot generates friction as it moves [1-3].

The orthodontic archwire and bracket's frictional contact may hinder or reduce the effectiveness of the desired tooth movement [4]. In order to get the best possible orthodontic results, this poses a major clinical problem in sliding mechanics and calls for careful treatment [5,6]. Several factors influence the amount of friction that develops at the point where the archwire and orthodontic bracket slot meet. These include the archwires' and brackets' material types and size, the angle at which the two are relative to one another, and accumulation of salivary deposits or debris on the archwire [7]. Reducing anchoring loss and improving orthodontic tooth movement efficiency are both achieved by minimizing frictional resistance during canine retraction. Crucially, a thorough understanding and control of friction are essential to avoid unintended tooth movements.

Stainless steel archwires, though widely used due to their strength and formability, are associated with relatively high friction levels, prompting a search for innovative methods to enhance their performance. Various approaches are tended to minimize the frictional resistance associated with stainless-steel orthodontic appliances [8]. Alongside these mechanical approaches, surgical acceleration procedures such as piezocision and flapless corticotomy have also been investigated to shorten treatment time [9]. Systematic reviews further highlight the comparative effectiveness of surgical versus non-surgical acceleration, reinforcing the importance of evaluating alternative, less invasive strategies [10].

One such method involves coating the orthodontic archwire with nanoparticles, which alters the surface characteristics and enhances both the mechanical and biological properties of the metallic material [11]. Nanocoating with titanium dioxide (TiO₂) offers antibacterial and anticorrosive properties while also reducing surface roughness [12]. Nanotechnology has emerged as a promising solution in this regard, particularly with the application of TiO₂ nanocoatings. Orthodontic wires with these coatings have better surface qualities, including less friction and roughness [13]. Clinical applications may benefit from more effective space closure and reduced treatment durations as shown in laboratory investigations comparing TiO₂-nanocoated archwires to traditional uncoated wires [14]. Additionally, biologic adjuncts such as platelet-rich plasma and platelet-rich fibrin have been reported to accelerate canine retraction, demonstrating the ongoing search for effective clinical accelerators [15]. Beyond biologic aids, systematic reviews of frictionless mechanics also emphasize the need for continuous exploration of strategies to improve sliding mechanics [16].

Therefore, the present in vivo split-mouth study was designed with the primary objective of comparing the rate of maxillary canine retraction between TiO₂ nanocoated and uncoated stainless steel archwire segments over four months.

## Materials and methods

Study design

This in vivo, split-mouth study was approved by the Institutional Review Board at Sri Siddhartha Dental College and Hospital, number SSDCHIEC/2023/48, dated April 18, 2023. Informed consent was obtained from all participants and their guardians prior to enrolment in the study.

Sample size calculation

The sample size was determined using G*Power version 3.1.9 (Heinrich-Heine-Universität Düsseldorf, Düsseldorf, Germany), based on data reported by Alfawal et al. [9]. In their randomized controlled trial, the primary outcome variable was the rate of canine retraction (mm/month), with a mean difference of approximately 0.5 mm/month between the intervention and control groups and a standard deviation of about 0.5 mm. Using these parameters, an effect size of 0.80, power of 80%, and α = 0.05 were applied, which indicated a minimum requirement of 12 participants. To account for potential dropouts and to strengthen the study’s statistical validity, we recruited 15 patients. Since an a priori power analysis was performed during planning, a separate post-hoc power analysis was not deemed necessary.

Randomization and blinding

Random allocation was performed using Microsoft Excel (Redmond, WA, USA) to determine which quadrant would receive the TiO₂-coated wire segment. The coated segment was randomly assigned to either the right or left maxillary quadrant in a split-mouth design. Allocation was concealed from both operator and patient. A designated assistant handled the allocation and archwire delivery. Outcome measurements were performed by a blinded examiner.

Participants, eligibility criteria, and settings

A total of 15 patients undergoing orthodontic treatment were recruited based on the following inclusion criteria: patients aged 14-30 years, with a full permanent dentition, requiring bilateral maxillary first premolar extraction as part of treatment for Class I bimaxillary protrusion, and eligible for canine retraction using standard MBT 0.022'' slot brackets. Exclusion criteria included the presence of active caries, periodontal disease, craniofacial anomalies, skeletal crossbite, occlusal interferences, and unilateral chewing habits. All patients received standard MBT 0.022'' slot prescription brackets. Initial alignment and levelling were completed before initiating canine retraction. Premolar extractions were performed two weeks prior to retraction mechanics.

Interventions and outcomes

**Figure 1:**
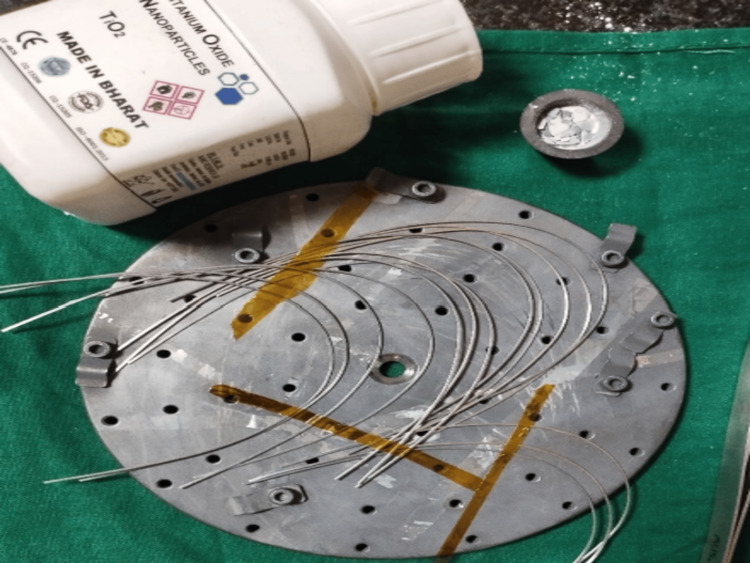
Titanium dioxide nanocoating on 19x25 inch stainless steel upper archwire 0.019 × 0.025-inch stainless steel archwire, where one half segment of the wire was coated with titanium dioxide (TiO₂) nanoparticles using the radiofrequency magnetron sputtering method and the other segment of the wire remained uncoated

Each participant received a 0.019 × 0.025-inch stainless steel archwire segment, in which one half of the segment was coated with TiO₂ nanoparticles using the radiofrequency magnetron sputtering method, as described by Liu et al. [17]. The coating was deposited under standardized parameters (sputter power 50-80 W, RF frequency 14.56 MHz, base pressure ~4 × 10⁻³ Torr, deposition pressure ~1 × 10⁻⁶ Torr, deposition rate 10 Å/s, target purity 99.9%), and the resulting TiO₂ layer thickness was confirmed by profilometry to be approximately 80-100 nm (Figure 1).

**Figure 2:**
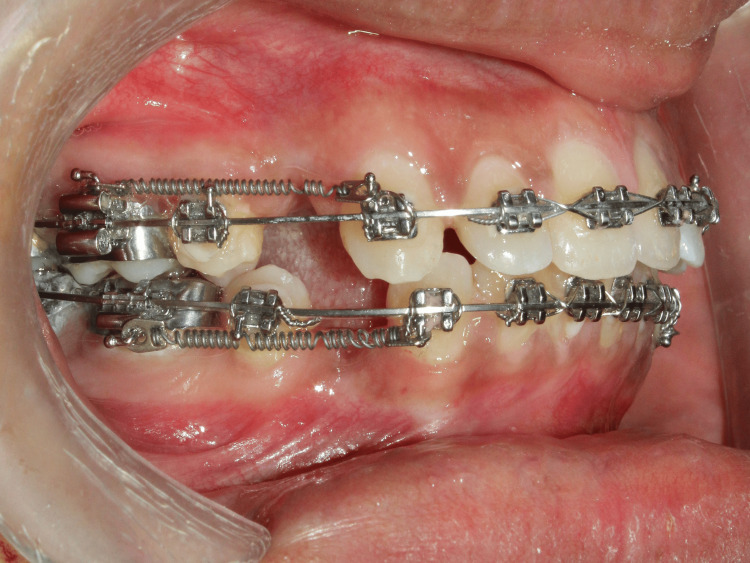
Right lateral view Intra-oral image of right lateral view where individual canine retraction with 19x25 inch stainless steel archwire using nickel-titanium closed coil springs of 150 grams of force given

**Figure 3:**
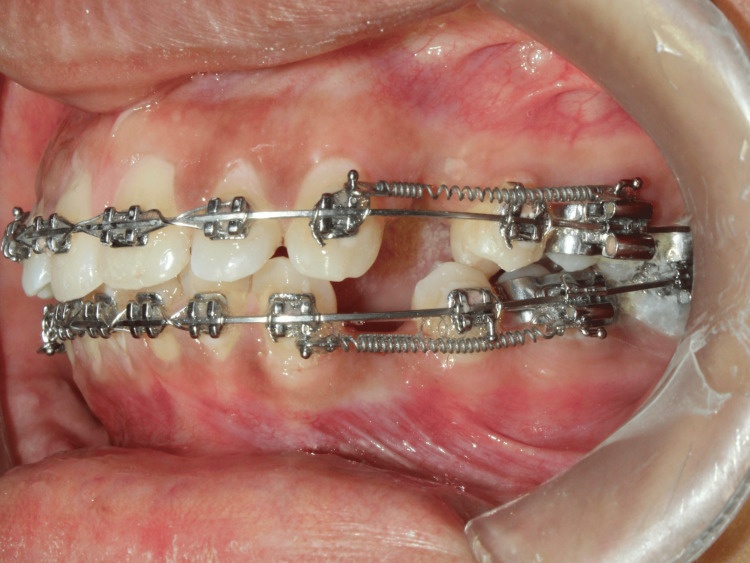
Left lateral view Intra-oral image of left lateral view where individual canine retraction with 19x25 inch stainless steel archwire using nickel-titanium closed coil springs of 150 grams of force given

Following wire placement, ligation was standardized using 0.010-inch stainless steel ligature wire. Retraction of the maxillary canine was performed using nickel-titanium closed-coil springs, calibrated to deliver a force of 150 g bilaterally from the canine hook to the molar tube. Force levels were checked and re-verified at each monthly appointment with a Dontrix gauge to maintain consistency, in line with recommendations for force monitoring in orthodontic trials by Jaber ST et al. [18]. Anchorage was reinforced using the second premolar, first molar, and second molar on each side. Care was taken to maintain not only biomechanical stability but also patient comfort, acknowledging that anchorage reinforcement strategies must balance mechanical control with patient-centered outcomes by Alfawal AM et al. (Figure 2, Figure 3).

**Figure 4:**
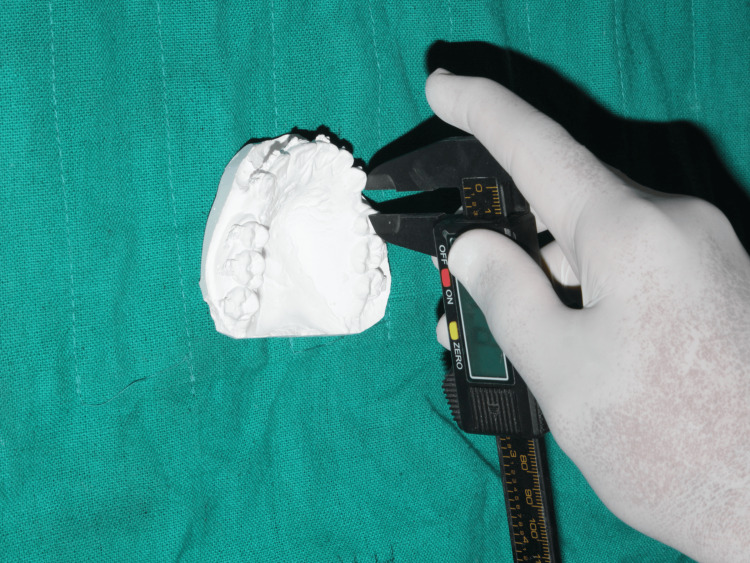
Rate of canine retraction measurement using digital vernier caliper on study models Measuring the rate of canine retraction using digital vernier caliper on study models from the distal surface of the upper canine to the mesial surface of the upper second premolar on both sides.

Impressions were taken with alginate and immediately poured in Orthokal at baseline (T0) and monthly intervals for four months (T1-T4). From the distal surface of the canine to the mesial surface of the second premolar, space was measured on the study models using digital Vernier calipers. The measurement points were explicitly defined to ensure consistency. To evaluate reliability, all measurements were repeated twice by the same examiner at a one-week interval. Intra-examiner reliability testing showed excellent agreement (ICC = 0.92), and the method error, calculated with Dahlberg’s formula, was 0.12 mm-well within the accepted standards for orthodontic measurements (Figure 4).

T0 is the amount of extraction space at the start of canine retraction; T1 is the rate of canine retraction and the amount of space closure at the end of the first month; T2 is the rate of canine retraction and the amount of space closure at the end of second month; T3 is the rate of canine retraction and the amount of space closure at the end of third month; and T4 is the rate of canine retraction and the amount of space closure at the end of fourth month. The rate of canine retraction was calculated as the difference in distance between timepoints.

Statistical analysis and data presentation

All measurements were recorded and analyzed using SPSS (version 20.0; IBM Corp., Armonk, NY, USA). The Shapiro-Wilk test was performed to assess normality at each timepoint. As some timepoints showed mild deviations from normality and the overall sample size was modest (n = 15), non-parametric tests were applied for hypothesis testing (Mann-Whitney U for between-side comparisons; Friedman test for within-group comparisons). A p-value < 0.05 was considered statistically significant.

## Results

**Figure 5:**
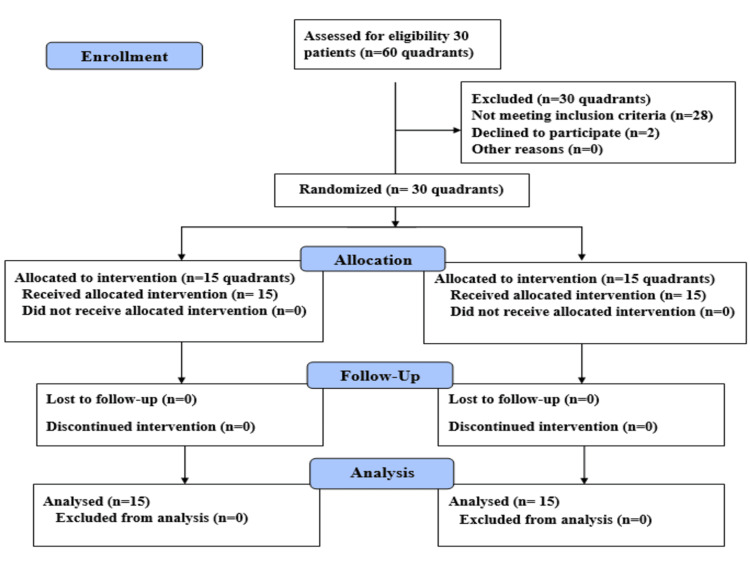
Consort flow chart of the patients throughout the trial

The procedure for patient selection and recruitment is outlined in Figure 5.

The rate of canine retraction at different time intervals is summarized in Table 1.

**Table 1:** Comparison of rate of canine retraction among the various groups at different time intervals All values are expressed as mean ± SD; Statistical test applied: #Mann-Whitney U test; ##Friedman test; Level of significance: p-value ≤ 0.05* is considered statistically significant.

Groups	Time intervals					^##^p- value
	T0	T1	T2	T3	T4	
Study group (n=15)	7.77 ± 0.15	5.99 ± 0.18	2.55 ± 0.21	0.47 ± 0.23	0.03 ± 0.08	<0.001*
Control group (n=15)	7.77 ± 0.15	6.17 ± 0.19	2.77 ± 0.37	0.69 ± 0.28	0.17 ± 0.19	<0.001*
^#^p- value	1.000	0.021*	0.126	0.021*	0.023*	

At baseline (T0), both groups showed identical values (7.77 ± 0.15 mm). Over time, a gradual reduction in space was observed in both groups.

At T1, the study group recorded a mean of 5.99 ± 0.18 mm, while the control group showed 6.17 ± 0.19 mm. The study group levels dropped even further to 2.55 ± 0.21 mm by T2, whereas the control group values dropped to 2.77 ± 0.37 mm. By T3, the distance had shrunk to 0.47 ± 0.23 mm for the study group and 0.69 ± 0.28 mm for the control group. By the fourth time point, very little room remained in both groups; the study group had 0.03 ± 0.08 mm while the control group had 0.17 ± 0.19 mm.

From T0 through T4, there was a consistent decline in values for within-group changes with time, suggesting that space was gradually closing as shown in Table 2.

**Table 2:** Pairwise comparison of rate of canine retraction within the various groups at different time intervals All values are expressed as mean differences; Statistical test applied: Wilcoxon-signed rank test; Level of significance: p-value ≤ 0.05* is considered statistically significant.

Time intervals	Study group		Control group	
	Mean difference	p-value	Mean difference	p-value
T1-T0	1.77	<0.001*	1.60	<0.001*
T2-T0	5.21	<0.001*	5.00	<0.001*
T3-T0	7.30	<0.001*	7.07	<0.001*
T4-T0	7.74	<0.001*	7.60	<0.001*
T2-T1	3.44	<0.001*	3.40	<0.001*
T3-T1	5.53	<0.001*	5.47	<0.001*
T4-T1	5.97	<0.001*	6.00	<0.001*
T3-T2	2.09	<0.001*	2.07	<0.001*
T4-T2	2.53	<0.001*	2.60	<0.001*
T4-T3	0.44	<0.001*	0.53	<0.001*

Values in the study group fell about 7.74 mm and in the control group about 7.60 mm between the first and last time periods, which is the most significant decrease.

**Figure 6:**
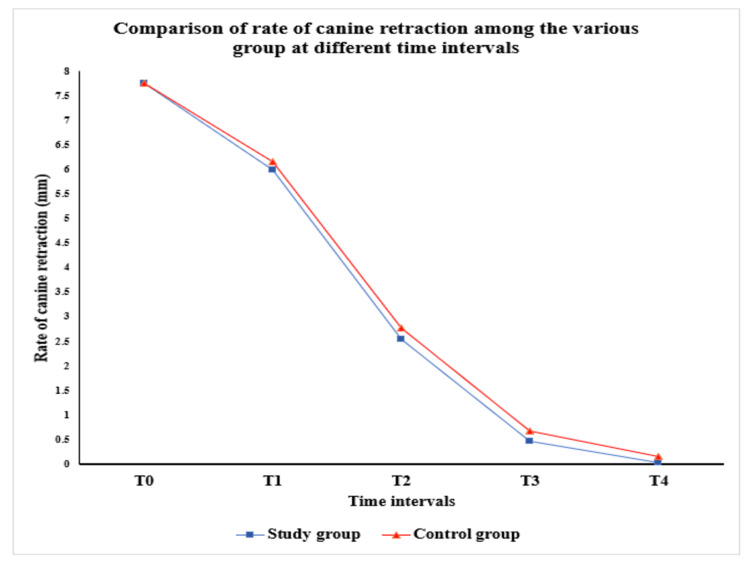
Comparison of rate of canine retraction among the various groups at different time intervals Visual representation showing consistently lower values in the study group at T1, T2, T3, and T4

Figure 6 visually represents these trends, showing consistently lower values in the study group at T1, T2, T3, and T4. This suggests improved retraction efficiency in the study group across the evaluated period.

## Discussion

Recent developments in nanotechnology have shown that altering the surface characteristics of orthodontic equipment might be a viable option for improving the efficacy of orthodontic therapy [19,20]. An example of a technological advancement is the coating of stainless steel archwires with nanoparticles of titanium dioxide [21]. The goal of these coatings is to decrease friction between the wire and the bracket, which might lead to faster tooth movement, more patient comfort, and a shorter treatment time [22]. In this in-vivo split-mouth research, the purpose was to compare the rate of canine retraction during orthodontic treatment using stainless steel archwire segments coated with TiO₂ on one side and those that were not coated on other side.

At four different time points (T0, T1, T2, T3, and T4), the study models were measured using a digital Vernier caliper to determine the canine's retraction. To compare each group across different time periods, statistical analyses were performed using the Friedman test.

When observing the rate of canine retraction, a consistent trend toward faster movement on the TiO₂ nanocoated side was evident. Specifically, notable variations were detected at the first, third, and fourth months (T1: p=0.021, T3: p=0.021, and T4: p=0.023) (refer to Table 1). No variations at baseline (T0: p=1.000) or during the second month (T2: p=0.126), suggesting that the beneficial effect of nanocoating became more pronounced as treatment progressed. Within-group comparisons using the Friedman test revealed notable variations (p<0.001) for both groups, indicating that space closure progressed consistently over time.

Further post-hoc pairwise analysis (as presented in Table 2) revealed substantial variations across all time intervals (p<0.001), highlighting the natural progression of canine retraction. The nanocoated segment showed a marginally faster rate of space closure compared to the other group (Figure 6). These outcomes align with previous studies that emphasize the value of TiO₂ nanocoatings. 

Kachoei et al. observed that physical vapor deposition (PVD) of TiO₂ onto orthodontic wires not only improved surface smoothness but also led to reduced frictional forces, a key determinant of efficient orthodontic tooth movement. These results closely support the present findings, where enhanced canine retraction was observed on the nanocoated side [21].

Chaturvedi et al. provided additional insight by emphasizing the biological advantages of surface modifications, highlighting that TiO₂ coatings improved both mechanical properties and biocompatibility [23]. Liu et al. further strengthened this view, noting that nanocoated wires could achieve superior mechanical performance with lower plaque accumulation and reduced bacterial adhesion, thus maintaining appliance integrity for longer durations [17].

Recent studies have continued to reinforce these observations. A study by Golsha et al. demonstrated that TiO₂ nanocoating significantly decreased frictional resistance in both wet and dry conditions, proposing it as a viable method to enhance orthodontic treatment outcomes [24]. Additionally, Indumathi et al. indicated that nano-modified orthodontic materials exhibited better wear resistance and surface quality, leading to more consistent force application [25]. These contemporary findings provide robust support to the observations made in the present investigation.

While Keerthi et al. demonstrated that TiO₂ coating improved surface smoothness of nickel-titanium archwires and reduced Streptococcus mutans adhesion, confirming its biological safety and mechanical potential. In contrast, our split-mouth clinical study directly evaluated the rate of canine retraction, showing statistically faster tooth movement with coated wires under real treatment conditions. This gives a more nuanced, clinically relevant understanding of performance, showing that TiO₂ coatings are effective and that maintaining coating integrity is key to maximizing benefit [26].

Maliael et al. and Bacela et al. confirmed that TiO₂ nanocoating reduces friction and aids tooth movement, but their findings were mainly from in vitro or short-term models [27,28]. This in vivo split-mouth design extends these results by demonstrating consistent, statistically significant acceleration of canine retraction under clinical conditions. Similar findings were reported by Suresh et al. demonstrated in vitro that TiO₂-nanocoated stainless steel wires experienced visible peeling off after friction tests, which reduced their intended friction-reducing benefit and further research is to be done by altering the size of nanoparticles and thickness of the coating [29].

Shaadouh et al. [30] stated that biologically mediated interventions can produce greater acceleration effects compared with mechanical adjuncts. In contrast, the modest but significant differences observed in our study suggest that TiO₂ nanocoating may serve as a non-invasive adjunct rather than a primary acceleration technique.

Limitations

The most significant limitation was associated with the durability of the nanocoating. Over the four-month clinical period, signs of peeling and erosion of the TiO₂ layer were noted even when coating thickness was altered. This degradation may have reduced the low-friction benefit over time, thereby influencing the observed rates of canine retraction. We recognize that this coating instability poses a major challenge to its long-term clinical applicability; however, our findings suggest that periodic replacement of coated wires at each activation interval may help sustain efficiency gains. Despite degradation, the persistence of improved movement on the nanocoated side indicates that even partial preservation of the coating contributes positively to orthodontic mechanics. Although post-trial scanning electron microscopy evaluation of retrieved wires was not performed, the gradual reduction in clinical performance strongly suggested surface degradation. Nanocoating thickness was maintained within recognized biocompatibility limits but future studies should incorporate long-term biological monitoring to further validate safety.

Another limitation was the modest sample size (n=15), which provided adequate statistical power for this pilot but reduces generalizability. Patient compliance also posed challenges: broken brackets, dislodged nickel-titanium coil springs, and missed appointments introduced variability in force delivery, despite careful monitoring and reactivation at each visit. Measurement accuracy was another potential source of error. While calibrated digital Vernier calipers were employed and intra-examiner reliability was confirmed (ICC = 0.92; Dahlberg’s error = 0.12 mm), plaster model measurements remain less precise than digital 3D methods.

We further acknowledge that ancillary variables such as anchorage loss, root movement, and periodontal indices were not evaluated, limiting biological interpretability.

Overall, while statistically significant improvements in canine retraction were observed at selected intervals, the magnitude of acceleration was modest. This suggests that although TiO₂ nanocoating may enhance efficiency, future large-scale studies with advanced coating technologies, larger samples, and adjunct surface analyses are warranted to establish definitive clinical recommendations.

## Conclusions

TiO₂ nanocoating on stainless steel archwire segments may enhance the efficiency of canine retraction compared with uncoated segments in this in vivo split-mouth study. Although the overall difference in total space closure was modest, statistically significant improvements at selected intervals affirm its potential short-term clinical relevance. Importantly, these effects are most consistent with a friction-reduction mechanism rather than a biologic acceleration of tooth movement, and the observed degradation of the coating during intraoral use highlights durability as a key limitation. The persistence of improved retraction on the coated side, even with partial peeling, suggests that periodic renewal of coated wires at monthly activations may help sustain benefit. Compared to more invasive surgical or biologic adjuncts, TiO₂ nanocoating offers a modest but non-invasive alternative, supporting its role as an adjunctive strategy. Future studies with larger samples, advanced coating technologies, and longer-term follow-up are warranted to clarify its durability and clinical applicability.
